# Retinal and choroidal detachment following corneal wasp sting: a case report and literature review

**DOI:** 10.1186/s12348-025-00510-9

**Published:** 2025-07-15

**Authors:** Shaofei Xue, Jiang Yao, Daniel Hillarion Scotland, Yuanyuan Qi

**Affiliations:** 1 The Third People’s Hospital of Dalian, Dalian, China; 2 Liaoning Provincial Key Laboratory of Cornea and Ocular Surface Diseases, Dalian, China; 3 Dalian Medical University, Dalian, China

**Keywords:** Wasp sting, Corneal injury, Retinal detachment, Choroidal detachment, Ocular toxicity

## Abstract

**Purpose:**

Ocular injuries caused by wasp stings are rare but potentially devastating. This report describes a case of severe retinal and choroidal detachment secondary to corneal wasp envenomation, with a focus on pathogenesis and management challenges.

**Case presentation:**

A 63-year-old female sustained a left corneal wasp sting, progressing to corneal edema, hypopyon, retinal-choroidal detachment, and eventual light perception loss. Despite medical therapy (topical corticosteroids, antibiotics), the patient declined surgical intervention, ultimately resulting in evisceration.

**Conclusion:**

Wasp venom triggers synergistic neurotoxic and immune-mediated damage, emphasizing the necessity of individualized management. Clinicians should prioritize multimodal approaches to mitigate irreversible vision loss in such cases.

## Background

In urban environments, hymenopteran insects (e.g., bees and wasps) are relatively uncommon, resulting in a low incidence of ocular hymenopteran sting injuries in clinical practice [1, 2]. The prognosis of such injuries varies significantly depending on factors including insect species, anatomical site of envenomation, and venom toxicity, which collectively influence the extent of immunogenic or cytotoxic damage [1–6]. Among hymenopterans capable of inflicting ocular trauma, honeybees and wasps are the two primary categories implicated in human attacks [7, 8]. Notably, honeybees exhibit docile behavior and rarely initiate unprovoked aggression [9]whereas wasps demonstrate heightened hostility, likely attributable to their venom composition. Specifically, wasp venom contains elevated concentrations of kinins and mast cell degranulating peptides compared to bee venom, which may explain their propensity for unprovoked attacks.

## Case presentation

A 65-year-old female was transferred to our hospital 16 hours after a left eye injury from a wasp sting. The patient had no history of hypertension, diabetes or significant systemic or ocular diseases. Upon admission, her best-corrected visual acuity (BCVA) was 0.5 in the right eye (with lens opacity) and light perception (+) with inaccurate light projection in the left eye. Slit-lamp examination revealed conjunctival hyperemia and chemosis, diffuse corneal stromal edema with epithelial defects, and a central, full-thickness, needle-like wound that was self-sealed, without residual stinger or foreign body in the left eye. The anterior chamber showed hypopyon, and the lens and posterior segment were not visible. Intraocular pressure (IOP) was T+3 in the left eye and 15 mmHg in the right eye. Initial diagnoses included toxic injury of the left eye due to wasp sting, secondary glaucoma, endophthalmitis, and age-related cataract in the right eye. Emergency treatment was initiated with topical antibiotics, corticosteroids, cycloplegics, and IOP-lowering agents.

**Fig. 1:**
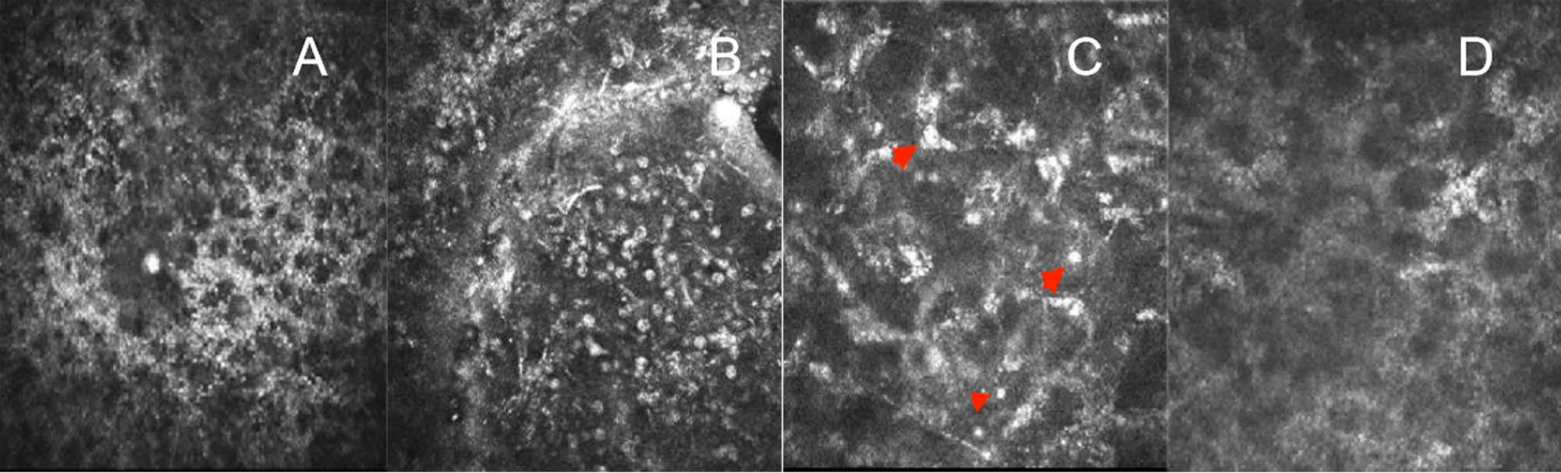
Day 3 post-injury: Confocal microscopy of the left eye cornea, (**A**) Disruption of the epithelial layer. **B** Needle-shaped, crystalline hyperreflective deposits are visible within the stroma. **C** Stromal infiltration with activated inflammatory cells and scattered hyperreflective particles (arrowheads). **D** Disorganization and loss of endothelial cells

**Fig. 2:**
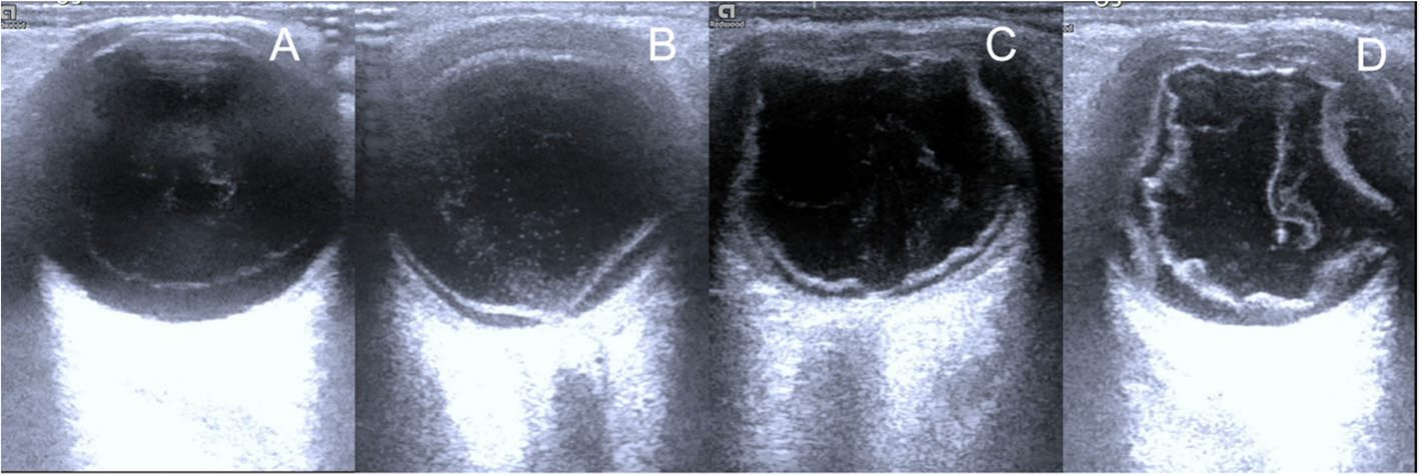
Ocular color ultrasonography of the left eye. **A**+**B** Day 3 post-injury: Retinal and choroidal detachment, indicating posterior segment involvement. **C**+**D** Day 30 post-injury: Marked globe atrophy with peripapillary hyperechoic lesions

On day 3 post-injury, the patient experienced clinical deterioration, presenting with no light perception (NLP) and hypotony (T-2). Slit-lamp examination revealed extensive corneal epithelial defects. Confocal microscopy confirmed stromal inflammation (Fig. 1). Ocular ultrasound confirmed retinal and choroidal detachment (Fig. 2 A+B), indicating posterior segment involvement. Consequently, additional diagnoses of left eye retinal and choroidal detachment were established. Treatment was intensified with topical steroids and adjunctive subconjunctival dexamethasone (3 mg/day). Systemic steroids and pars plana vitrectomy were recommended; however, the patient declined surgical intervention and was discharged.

On day 30 post-injury, the patient was admitted to the hospital again due to severe and unbearable pain in the left eye. Examination showed NLP, corneal melting (3 × 4 mm central ulceration), and peripheral scleral thinning. Ocular ultrasonography revealed an atrophic globe with peripapillary hyperechoic lesions (Fig. 2 C+D). The patient opted against further conservative treatment and underwent left eye evisceration with orbital implant placement.

Three months after the injury (two months after the left eye surgery), the patient underwent re-examination. The condition of the left eye remained stable post-evisceration. Visual acuity in the right eye remained unchanged, and there were no other systemic or ocular complications.

### Discussion and conclusions

Based on this case, our review aims to comprehensively discuss the pathogenesis, clinical manifestations, diagnostic approaches, and therapeutic strategies for ocular injuries caused by hymenopteran stings, thereby advancing the understanding of their management. The injury mechanisms of hymenopteran stings encompass: (1) direct mechanical trauma from the sting, (2) toxic effects of venom, and (3) host immune responses to venom components [3, 10, 11]. Notably, the barbed stinger of honeybees (e.g., Apis mellifera) often detaches and remains embedded in human tissues during envenomation, along with the venom apparatus and sac. Subsequent attempts to remove the stinger may inadvertently exacerbate injury through additional venom release or mechanical disruption [5, 9–11]. In contrast, wasp stingers are smooth and rarely retained, minimizing secondary trauma [9, 11]. 

The venom of Hymenopteran insects is a complex mixture containing three primary categories of bioactive components: (1) peptides (melittin, apamin, and mast cell degranulating peptides, (2) biogenic amines (histamine, serotonin, acetylcholine, and dopamine), and (3) enzymes (hyaluronidase, phospholipase A, and phospholipase B) [6, 12–14]. Melittin, the most abundant (50–60%) and potent toxin in wasp venom, disrupts lipid bilayers, causing cell membrane lysis, hemolysis, and lens protein denaturation, which may contribute to cataract formation [2, 11, 15, 16]. Apamin, accounting for 1–3% of venom, selectively inhibits Ca²⁺-dependent K⁺ channels in the central nervous system, leading to neurotoxic effects such as intraocular muscle paralysis (via third cranial nerve dysfunction), sectoral iris palsy, optic neuritis, and optic atrophy following corneal stings [10, 11, 15].

Biogenic amines like histamine and acetylcholine mediate vasodilation, hyperemia, and edema [2, 6, 13]while dopamine and other amines exacerbate localized pain and swelling [4, 17]. Hyaluronidase (1–3%), an enzyme shared with other animal venoms, facilitates venom spread by degrading hyaluronic acid in the extracellular matrix and increasing capillary permeability [9, 15]. Phospholipase A2 (10–12%), the second most abundant compound in bee venom, is a potent allergen that synergizes with melittin to enhance membrane phospholipid degradation [9]. Although phospholipase A2 alone is non-toxic, its complexation with melittin amplifies cytotoxic effects [9]. High-molecular-weight enzymes such as phospholipases A/B and hyaluronidase degrade anterior iris pigment cells, resulting in heterochromia [2]. These enzymes also exhibit strong antigenicity, eliciting IgE-mediated type I hypersensitivity reactions [18].

Ocular hymenopteran stings introduce a complex venom mixture into the eye, eliciting both toxic and immune-mediated responses that culminate in vision-threatening complications [19] such as corneal opacity, bullous keratopathy, endothelial cell damage, cataract, glaucoma, uveitis, and optic neuropathy [2, 5, 16, 20–22]. Long-term follow-up evaluations are imperative to monitor potential late-onset complications [23]. The cornea is the most frequently affected site [3, 6, 16, 24, 25] likely due to its direct exposure and critical role in visual function. Studies indicate that hornet venom sprayed onto the ocular surface can induce corneal damage, including neuropathic pain and keratitis [26]. For instance, Takashi et al. reported two cases of severe corneal endothelial injury caused solely by hornet venom spray—without direct stinging—highlighting the toxin’s capacity to penetrate and damage ocular tissues [3]. Similarly, Vuslat Pelitli documented a patient with persistent corneal endothelial cell density loss one year after a honeybee sting, suggesting long-term cytotoxicity of bee venom [27]. Wasp stings often provoke corneal edema, which may progress to bullous keratopathy.

Venom components also trigger immune-inflammatory cascades, leading to uveitis, hyphema, and elevated intraocular pressure [18]. Furthermore, the potent neurotoxicity of venom can induce toxic optic neuropathy, manifesting as optic neuritis, optic disc edema, and subsequent optic atrophy, ultimately causing severe vision loss or blindness [18]. Early diagnosis of neuro-ophthalmic complications is critical, as delayed intervention correlates with poor prognosis. Intravenous methylprednisolone may improve visual recovery if administered promptly [18].

Notably, visual impairment following stings is not always secondary to direct ocular injury. Systemic envenomation, particularly involving the head or neck, can also precipitate optic neuropathy. For example, Lee YS et al. described a patient stung on the right hand by a honeybee who developed blurred vision and optic disc edema in the right eye three days post-injury. Follow-up at three months revealed bilateral thinning of the retinal nerve fiber layer in the nasal and temporal quadrants [17]. Maltzman et al. summarized six cases of optic neuropathy after hymenopteran stings, with only one case involving direct ocular envenomation; the remainder involved stings to the head or neck. All patients exhibited localized cutaneous reactions followed by vision loss, optic disc edema, hyperemia, and peripapillary hemorrhages [28].

The diagnosis of hymenopteran stings is relatively straightforward and relies on a typical sting history combined with slit-lamp examination, which allows direct visualization of the sting site and penetration depth [21]. A comprehensive ocular examination is essential, including eyelid eversion to inspect the conjunctival fornices for retained foreign bodies [29]. Particular attention should be paid to eyelid and tarsal plate screening in cases of eyelid stings, as these injuries are prone to underdiagnosis and may lead to delayed treatment [21].

In suspected microbial keratitis, corneal scrapings should be collected for smear and culture to identify the causative organism [30]. Gudiseva et al. emphasized the necessity of microbiological analysis of extracted stingers to rule out secondary infections [2]. Distinguishing between sterile and infectious endophthalmitis remains a significant challenge when severe anterior segment reactions are triggered by toxic substances [13, 24]. Mohit Dogra et al. highlighted that microbiological analysis of vitreous samples is instrumental in differentiating sterile from infectious endophthalmitis in the context of severe anterior segment inflammation [24]. Advanced imaging modalities, such as anterior-segment optical coherence tomography (AS-OCT) and in vivo confocal microscopy (IVCM), are valuable for delineating lesion depth, assessing inflammatory severity, and detecting residual foreign bodies. For instance, Yuen et al. utilized confocal microscopy to identify residual insect-derived particles in the corneal stroma of a bee sting patient, with stable vision and no particle migration observed over a six-month follow-up period [31]. The high-resolution optical capabilities of confocal microscopy enhance the detection of subtle stromal foreign bodies [31]. Additionally, corneal endothelial specular microscopy plays a crucial role in long-term follow-up by quantifying endothelial cell loss and monitoring corneal decompensation [11].

Hymenoptera stings to the ocular region may provoke complex toxic and immune-mediated inflammatory reactions, necessitating individualized therapeutic regimens contingent upon clinical severity [23]. Current management strategies are broadly categorized into medical therapy and surgical interventions. First-line pharmacological treatments include topical antibiotics, corticosteroids, antihistamines, and cycloplegics [21, 25]. Khalid et al. recommend initiating broad-spectrum antibiotics empirically, followed by targeted therapy based on culture results [10]. González et al. reported a case of secondary keratitis caused by a wasp sting. The patient was treated with early intensive corticosteroids (systemic and topical application) and antibiotics, achieving significant visual recovery with stable outcomes during long-term follow-up [23]. Topical corticosteroids are pivotal for mitigating inflammation, while antihistamines address immune-mediated responses [10]. For severe corneal involvement or intense anterior chamber reactions, systemic corticosteroids may supplement topical regimens to prevent complications such as endothelial decompensation, cataract, and glaucoma [2, 6]. However, Ono et al. caution against corticosteroid use in cases of culture-proven secondary bacterial infections, prioritizing infection control [3].

The acute inflammation following hymenopteran stings likely arises from a combination of mechanical trauma, venom toxicity, and immune reactions, which complicates the decision to remove retained stingers. Current evidence on stinger extraction remains limited to case reports, and the optimal timing for removal remains controversial in clinical practice [1].

The most widely accepted approach for insect-derived foreign bodies involves prompt removal after confirming no intraocular penetration, thereby minimizing venom release, suppressing exogenous inflammatory triggers, and reducing complication risks [1, 4, 6, 12]. Visscher suggested that it should be removed as soon as possible; the amount of venom injected correlates directly with the duration of the stinger’s retention [32]. Immediate extraction is strongly recommended if the stinger is easily accessible or threatens the visual axis [1, 6]. However, aggressive removal of deeply embedded corneal stingers risks iatrogenic venom leakage or corneal wound exacerbation, necessitating cautious handling [6, 11]. Tyagi et al. demonstrated the efficacy of endoscope-assisted stinger removal, which enhances precision and minimizes collateral damage to adjacent ocular structures [25].

Conversely, stingers without active venom release may act as inert bodies and remain stable within the cornea over the long-term, particularly when located away from critical zones. Conservative management is thus justified in such cases [1, 6, 10]. For example, Rai et al. reported a patient with a non-penetrating corneal stinger who maintained stable vision over 16 months of observation, with no stinger migration or complications [1].

Patients with ocular wasp or bee stings may develop a spectrum of complications, some of which necessitate surgical intervention. These procedures include phacoemulsification with intraocular lens implantation [2, 20, 22, 25] penetrating keratoplasty [22] Descemet-stripping automated endothelial keratoplasty [3, 11, 20, 25] trabeculectomy [2, 3]and pars plana vitrectomy [13, 24].

In this case, the patient developed endophthalmitis, retinal detachment, and choroidal detachment, likely resulting from a hypersensitivity reaction to wasp venom, compounded by synergistic effects of inflammatory cytokine release, increased intraocular capillary permeability, and protein exudation. Subsequent loss of light perception may also reflect concurrent optic neurotoxicity induced by venom components. Although rare, ocular hymenopteran stings can cause catastrophic visual outcomes, requiring a systematic clinical approach. This includes comprehensive history-taking to clarify injury context (e.g., insect species, time of envenomation, systemic symptoms), multimodal imaging (ultrasound, OCT, confocal microscopy) to evaluate structural integrity and detect retained foreign bodies, immediate medical therapy (topical/systemic corticosteroids, antibiotics) to counteract venom toxicity and inflammation, and long-term surveillance for complications such as glaucoma, corneal decompensation, or optic atrophy.

## Data Availability

No datasets were generated or analysed during the current study.
