# Correction: Retinal and choroidal detachment following corneal wasp sting: a case report and literature review

**DOI:** 10.1186/s12348-025-00528-z

**Published:** 2025-11-28

**Authors:** Shaofei Xue, Jiang Yao, Daniel Hillarion Scotland, Yuanyuan Qi

**Affiliations:** 1 The Third People’s Hospital of Dalian, Dalian, China; 2 Liaoning Provincial Key Laboratory of Cornea and Ocular Surface Diseases, Dalian, China; 3 Dalian Medical University, Dalian, China

**Correction: J Ophthalmic Inflamm Infect 15, 53 (2025)**

**https://doi.org/10.1186/s12348-025-00510-9**

Following publication of the original article, it came to the authors' attention that in the section Case Presentation, the article had published with outdated versions of the figures and, furthermore, the the figures were incorrectly order. The figures have now been corrected in this section. Thank you for reading this erratum.

